# Characterization of *Burkholderia cepacia* Complex Core Genome and the Underlying Recombination and Positive Selection

**DOI:** 10.3389/fgene.2020.00506

**Published:** 2020-05-21

**Authors:** Jianglin Zhou, Hongguang Ren, Mingda Hu, Jing Zhou, Beiping Li, Na Kong, Qi Zhang, Yuan Jin, Long Liang, Junjie Yue

**Affiliations:** ^1^State Key Laboratory of Pathogen and Biosecurity, Beijing Institute of Biotechnology, Beijing, China; ^2^Institutes of Physical Science and Information Technology, Anhui University, Hefei, China

**Keywords:** *Burkholderia cepacia* complex, recombination, positive selection, COG, core genome

## Abstract

Recombination and positive selection are two key factors that play a vital role in pathogenic microorganisms’ population adaptation and diversification. The *Burkholderia cepacia* complex (Bcc) represents bacterial species with high similarity, which can cause severe infections among cases suffering from the chronic granulomatous disorder and cystic fibrosis (CF). At present, no genome-wide study has been carried out focusing on investigating the core genome of Bcc associated with the two evolutionary forces. The general characteristics of the core genome of Bcc species remain scarce as well. In this study, we explored the core orthologous genes of 116 Bcc strains using comparative genomic analysis and studied the two adaptive evolutionary forces: recombination and positive selection. We estimated 1005 orthogroups consisting entirely of single copy genes. These single copy orthologous genes in some Cluster of Orthologous Groups (COG) categories showed significant differences in the comparison of several evolutionary properties, and the encoding proteins were relatively simple and compact. Our findings showed that 5.8% of the core orthologous genes strongly supported recombination; in the meantime, 1.1% supported positive selection. We found that genes involved in protein synthesis as well as material transport and metabolism are favored by selection pressure. More importantly, homologous recombination contributed more genetic variation to a large number of genes and largely maintained the genetic cohesion in Bcc. This high level of recombination between Bcc species blurs their taxonomic boundaries, which leads Bcc species to be difficult or impossible to distinguish phenotypically and genotypically.

## Introduction

Two evolutionary forces are important for the abilities of microorganisms to adapt to their novel immune systems, antibiotics, and hosts. One is positive selection, which favors the fixation of advantageous mutations, and the other is recombination, which helps microorganisms acquire new genetic material. Recent studies suggest that these two processes play a vital role in genomic adaptive evolution in microorganisms. To be specific, the genomes in microorganisms are classified as accessory genome (in which genes exist in certain species strains alone), and core genome (in which the comprising genes exist in all species strains) (Tettelin et al., 2005). Of them, the latter is considered as the typical bacterial taxa at a variety of taxonomic levels (Ochman et al., 2005), and its adaptive changes are clearly important in the evolution of bacteria (Lefébure and Stanhope, 2007; Xu et al., 2011; de Been et al., 2013). Genes at positive selection pressure have been identified from core genomes of *Salmonella enterica* and *Escherichia coli* through evolution analyses at molecular level (Charlesworth and Eyre-Walker, 2006; Chen et al., 2006). Additionally, positive selection in genes of pathogenic agents like *Actinobacillus pleuropneumoniae* (Xu et al., 2011), *Campylobacter* (Lefébure et al., 2010), *Pasteurella multocida* (Cao et al., 2017), and *Listeria monocytogenes* (Tsai et al., 2011) is also reported. The above-mentioned positive selection examples reveal its vital role in the adaptation of various pathogens to a variety of environmental niches, which is achieved through the escape from immune response in the host and the optimization of infection process. Moreover, recombination has been identified as the other vital parameter in pathogen adaptation, and bacterial recombination rates possibly increase relative to the mutation rates (Awadalla, 2003; Hao and Golding, 2006). Additionally, recombination allows for inter- and intra-species genetic material exchange, which facilitates to maintain the population structures and homogenize the core genome (Gonzalez-Torres et al., 2019). Furthermore, in some bacteria such as *Helicobacter pylori*, *Streptomyces*, and *Neisseria* (Suerbaum et al., 1998; Doroghazi and Buckley, 2010; Kong et al., 2013), recombination occurs frequently and can possibly accelerate their adaptation (Cooper, 2007).

The Bcc has been recognized to be a controversial group of those Gram-negative bacteria that are constituted by ≧22 effective species (Devanga Ragupathi and Veeraraghavan, 2019). Species belonging to the Bcc are opportunistic pathogens that have been involved in chronic and the severe infections among cases suffering from the chronic granulomatous disorder and cystic fibrosis (CF) (LiPuma, 2010). Outbreaks caused by other Bcc species have occurred worldwide, and patient-to-patient spread has been reported (Mahenthiralingam et al., 2008). In recent years, Bcc are generally regarded as the most harmful CF pathogens and have been shown to be of great concern for patients and manufacturers of drugs and products that contribute to patient health. Many studies have focused on the identification and taxonomy of Bcc due to the likeness between different Bcc bacteria (Papaleo et al., 2010; Bach et al., 2017; Devanga Ragupathi and Veeraraghavan, 2019). Bcc also exhibit high diversity. According to a comparative analysis of *Burkholderia cenocepacia*, about 21% genome shows uniqueness compared with additional strains of *B. cenocepacia*, which also emphasizes the genomic plasticity within Bcc species (Holden et al., 2009). Bcc has a large genome consisting of multiple circular chromosomal replicons, containing twice the amount of genetic material as *E. coli*. Bcc species encode many drug- and virulence-resistant genes and extensive functions with metabolic versatility (Shommu et al., 2015; Rhodes and Schweizer, 2016; Sousa et al., 2017), which allow them to adapt to a wide range of environments (Mahenthiralingam et al., 2005; Eberl and Vandamme, 2016).

Though there is a wealth of knowledge describing the taxonomic status, pathogenicity and genomic properties of the Bcc microorganism, information regarding their adaptive evolution, such as positive selection and recombination, have not been clearly elucidated. So far, the existing whole-genome articles do not investigate those genes associated with two Bcc bacterial evolutionary forces as well as corresponding core genomic contents, and the related Bcc species features are rarely investigated. To date, an increasing number of complete genome sequences of the bacteria in the Bcc have been made publicly available^1^. In this work, genome sequences in Bcc strains were used to employ comparative genomics to elucidate the core genome content of Bcc and related adaptive evolution. We inferred single copy orthologous genes of Bcc, and they shared one common ancestor through speciation. Associations of genes with evolutionary features, such as *d*_N_, *d*_S_, nucleotide diversity, and codon bias, were determined through statistical tests. We conducted a comprehensive genome-wide scan to investigate genes that undergo recombination and positive selection during Bcc’s evolution. Our study will gain insight into the genome dynamics of Bcc species.

## Materials and Methods

### Dataset Preparation and Identification of Orthologous Genes

A total of 116 genomes of Bcc strains were used in this study. They were carefully selected for quality and representativeness. To be specific, all available assemblies of Bcc whole genomes in Complete Genome, Chromosome, and Scaffold levels were downloaded from the GenBank database (as on April 14, 2019) (Sayers et al., 2019). Their quality was estimated by running CheckM with the lineage-specific workflow and default parameters (Parks et al., 2015). A genomes was excluded if it failed to satisfy the requirement of ≥90% completeness, ≤10% contamination and an overall quality ≥50% (defined as completeness – 5 × contamination) (Parks et al., 2018). After filtering, the genomes were further dereplicated according to their pairwise average nucleotide identity (ANI) values that were determined by FastANI (Jain et al., 2018), as described in Parks et al. (2017). One hundred and twelve Bcc genomes were kept for subsequent analysis after quality checking and dereplication. Addtionlly, four Bcc genomes that were Contig-level assemblies were added to our dataset because they were assembled from type material and their species did not have any better assembly sequences from the type strain. It came to a grand total of 116 genomes in our dataset. Groups of orthologous sequences were defined using OrthoFinder2 (version 2.3.3) (Emms and Kelly, 2019) and aligned with MAFFT version v7.271 (Katoh and Standley, 2013). Single copy genes present in all genomes were defined as the core orthologous genes and were used in the subsequent analyses. All of them had at least 50 codons. Detailed information for the analyzed genomes is listed in Supplementary Table S1.

### Calculation of Informative Sites, Nucleotide Diversity, dS, dN, and Codon Bias

The single copy orthologous protein sequence alignments and their open reading-frame nucleotide sequences were used to obtain corresponding multiple codon alignments using PAL2NAL (Suyama et al., 2006). Based on the multiple codon alignments, the PhiPack program was used to calculate nucleotide diversity and informative site number of every single copy core gene (Bruen et al., 2006). In addition, the numbers of synonymous (*d*_S_) and nonsynonymous (*d*_N_) substitutions per site were predicted for all single copy genes according to the SNAP method (Nei and Gojobori, 1986; Korber-Irrgang, 2000). The valid codon number (N_c_), which ranges from 20 with the most potent bias to 61 with non-bias (Wright, 1990), was used to measure the codon usage variation in every core gene. It was estimated by the program CodonW^2^.

### Functional Annotation and Subcellular Localization

Functional categories for every gene were assigned using the eggNOG-mapper v2 online service^3^ with minimal 50% query coverage and other default parameters (Huerta-Cepas et al., 2019). For each family (orthologous group), when over 55% genes shared an identical EggNog annotation, then, such specific EggNog annotation would be provided for this family; otherwise, this family was recognized to have an unclear function. Additionally, we used the curated part of the Pfam database (Pfam-A) release 31 and HMMER (version 3.1b2) to assign the Pfam domains to every sequence for each orthologous group (Eddy, 2011; Finn et al., 2014). The alignments of domain families of type *repeat* or *motif* were excluded because it is much harder to estimate their phylogeny (Forslund et al., 2008). Only the alignments with at least 60% coverage of Pfam-A families were kept. The subcellular localization of the interested proteins were predicted using BUSCA (Bologna Unified Subcellular Component Annotator) with the “Prokarya-Gram-negative” method (Savojardo et al., 2018).

### Identification of Composite Genes

The all-versus-all BLAST (blastp version 2.2.31+) was used to search homologies among the amino acid sequence pairs of all single copy genes; meanwhile, the threshold E-value was 1e-5, the soft masking parameter was set, and the max target sequences was 5000 (the others by default) (Altschul et al., 1997). The BLAST results were input to program CompositeSearch to deduce composite genes, and the threshold default identity was set at 30%, while the overlaps of amino acids were set at 20 for limiting the false negative error (Pathmanathan et al., 2018).

### Detection of Homologous Recombination

The homologous recombination signatures were searched in single copy core genome of Bcc using four different statistical procedures: GENECONV (Sawyer, 1989), pairwise homoplasy index (PHI) (Bruen et al., 2006), maximum χ^2^ (Smith, 1992), and neighbour similarity score (NSS) (Jakobsen and Easteal, 1996). For GENECONV, we used a gscale parameter of 1 to allow mismatches within recombining fragments and otherwise default settings. In addition, for those putative recombinant areas, their significance was indicated using inner fragments *P*-values of 10,000 random permutations. Meanwhile, the PhiPack package (Bruen et al., 2006) was applied to implement the rest 3 procedures, which were run with the **−p** option and other default parameters.

### Detection of Positive Selection

The positive selection evidence for every single copy gene was tested according to maximum likelihood (ML), while the PAML 4.9 **codeml** program was applied to deduce those sites selected positively (Yang, 2007) according to previous description (Yu et al., 2016). Maximum likelihood phylogenies were inferred for each gene of the single copy core genome of Bcc using FastTree version 2.11 with multi-threaded and parameters “-nosupport -gtr -gamma -spr 4 -mlacc 2 -slownni -nt” (Price et al., 2010). The resulting topologies of ML trees were used as one of the inputs of the **codeml** program. A total of two site-specific models pairs were applied for analyzing every core orthologous family, namely, M2a (PositiveSelection) vs. M1a (NearlyNeural), as well as M8 (Beta&ω) vs. M7 (Beta), and they were different in terms of ω ratio (ω = *d*_N_/*d*_S_) assumed statistical distribution. The extra class of sites with partial sites screened positive that had ω > 1 was added into the alternative M2a model, whereas only site classes that had ω of 0–1 were allowed in the null model M1a; with regard to the other M8 alternative model, the additional sites screened positively that had ω > 1 were added, while for the M7a nested null model, ω was assumed for beta-distribution across various sites (Yang et al., 2000; Wong et al., 2004). In addition, M1a was compared with M2a, whereas M7 was compared with M8, to deduce the positively selected amino acid sites through the likelihood ratio test (LRT). Moreover, the *P*-value was measured based on LRT score that was determined via the PAML package module χ^2^ using 2 degrees of freedom (DOF). The approach was employed to estimate the Later, posterior probability for those sites under positive screening pressure was estimated through Bayes empirical Bayes (BEB) method according to the likelihood framework (Yang et al., 2005).

### Pan- and Core-Genome Analysis

Comparative genomics analysis results were obtained from OrthoFinder (Emms and Kelly, 2019). The gene count of all orthogroups was converted to a 0/1 matrix (also called pan-matrix) where, rows represent orthogroups and columns represent the genomes. For each orthogroup, a “1” means the presence of certain gene from respective genome while a “0” means absence. The binary matrix was input into PanGP software to generate the gene accumulation curves of pan- and core-genome using Distance Guide method, 5000 sample size, 100 sample repeat, and 100 amplification coefficient (Zhao et al., 2014). The expansiveness of pan-genome was estimated using micropan package in R with Heap’s law model and 1000 random permutations (Tettelin et al., 2008; Snipen and Liland, 2015). Additionally, a rooted species tree of Bcc was obtained from OrthoFinder and plotted with pan-matrix using phytools package (Revell, 2012).

### Soft-Core Orthogroups Analysis

Besides single copy gene clusters, orthogroups in soft-core genome were additionally analyzed. To increase the accuracy and power of our analysis, an orthogroup in the soft-core genome was removed if it meets any of the following criteria: minimal length of the protein sequences less than 50, the difference in protein lengths larger than 20%, more than one copy from each genome (Xu et al., 2011; Yu et al., 2016). The left orthogroups were subsequently performed on general characteristics calculation, functional annotation and recombination analysis, just as described above. Positive selection on these filtered orthogroups also was detected, except only the site-specific models pair M1a vs. M2a was performed due to the computational resources and time.

### Structure Modeling and Analysis

The three-dimensional structures for those orthologous genes screened under positive pressure were modeled using I-TASSER webserver (Yang and Zhang, 2015). Structural conformations, which were referred to as decoys, were generated through I-TASSER and clustered according to the similarity of sequence pairs. According to C-score, those top models had been screened to be the representative models for subsequent analysis. Those positively screened sites were then subjected to mapping to structure, and the PyMol software^4^ was used for visualization.

### Statistical Analysis

For controlling Type I errors, Benjamini–Hochberg’s method was used to correct multiple testing (Benjamini and Hochberg, 1995). In addition, the *q*-values were determined based on *P*-values by the use of R package QVALUE for all genes that had been tested for their positive screening and homologous recombination; meanwhile, the false discovery rate (FDR) was set at 10%, while the true null hypothesis proportion was preset at 1 (p0 = 1) (Storey and Tibshirani, 2003).

Differences across different features, such as nucleotide diversity, informative site number, codon bias, *d*_N_ and *d*_S_, were examined for their significance through nonparametric Mann–Whitney U-test between those residual Cluster of Orthologous Groups (COG) categories and the given COG. The binomial test was employed to determine the relationships of every COG with the evolutionary forces (positive screening and homologous recombination). With reference to the one-sided test number, Bonferroni corrections were carried out for multiple comparisons. Typically, the level of significance was determined at 5%. The R 3.6.1 (Team, 2013) and *in-house* Python scripts (Sanner, 1999) were applied for statistical analyses.

## Results

### Pan-Genome Profile and Phylogeny

**FIGURE 1:**
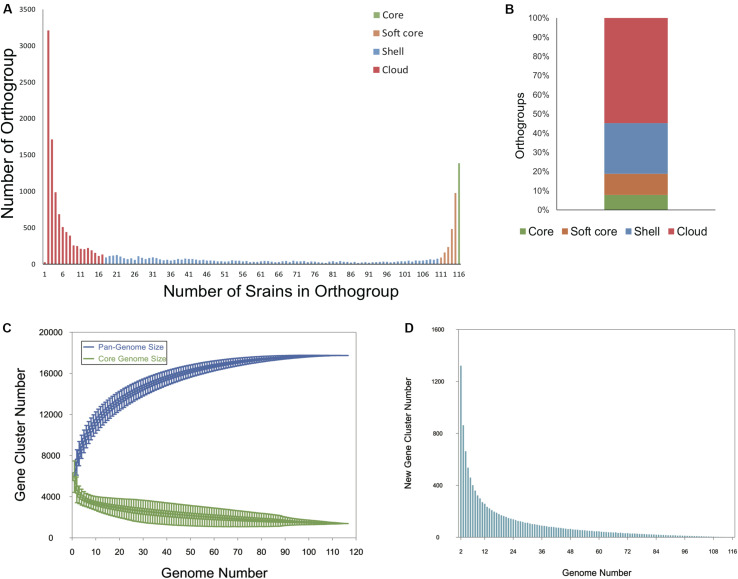
Pan -genome analysis of 116 Bcc whole-genome sequences. **(A)** Bar plot showing the frequencies of orthologous clusters. **(B)** Stacked bar plot of percentage of orthogroups in core, soft core, shell, and cloud genomes. **(C)** Gene accumulation curves of the Bcc pan- (blue) and core-genome (green). The estimation was made by including genomes one by one. **(D)** Quantitative relationship between new genes and sequenced genomes.

To determine the entire genomic repertoire of the Bcc population, a pan-genome analysis was performed based on the annotated protein sequences of the 116 strains. The 751,916 protein sequences present across all genomes were clustered into 17,740 orthogroups (i.e., the pan-genome) by OrthoFinder. Among them, 1387 orthogroups (7.82%) were conserved in all the 116 genomes, representing the universal core-genome of Bcc, and genes in 1005 orthogroups of universal core-genome kept only one copy in every strain; 1955 orthogroups (11.02%) constituted the soft-core genome (present in 95– < 100% of genomes); 4684 orthogroups (26.40%) were shell genes (present in 15–94% of genomes); and 9714 orthogroups (54.76%) were cloud genes that present in less than 15% of all the genomes (Figures 1A,B). Although cloud genes account for more than half of the pan-genome, only 27 (0.15%) orthogroups were singletons present in only one strain (Figure 1A). Figure 1C is a plot exhibiting the size of the pan-genome and universal core genome as functions of the number of randomly considered genomes. The core genome gradually converged to 1387 orthogroups as expected. Although the gene accumulation rarefaction curve of pan-genome seemed to reach a plateau after the inclusion of 110 genomes, the pan-genome showed characteristics of an “open” pan-genome (Tettelin et al., 2005). When taking the included genomes from 110 to 116, the size of the pan-genome increased from 17,735 to 17,740 (median value of the permutations). New genes continued to add to the pan-genome, even nine genes were contributed to the gene pool after the inclusion of No.116 genome (Figure 1D). What is more, the Heap’s law model parameter (α) was estimated to 0.96 by micropan (Snipen and Liland, 2015), which is less than the threshold of α = 1.0 that differentiates open from the closed genome. All told, our results suggest that Bcc holds an open pan-genome.

**FIGURE 2:**
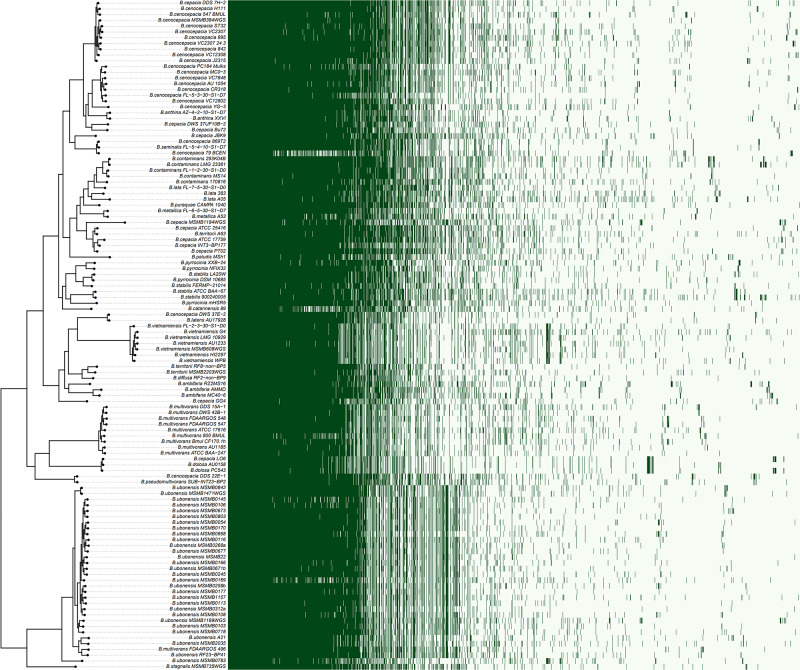
The species tree of Bcc against the genes presence/absence matrix. The species tree was constructed using STAG algorithm and rooted by STRIDE algorithm in OrthoFinder (Emms and Kelly, 2019). The heatmap on the right showed the presence (dark green) or absence (light green) of all 17,740 orthogroups. Each row in the matrix corresponds to a branch on the tree (i.e., one genome) and each column represented an orthogroup.

A rooted species tree of Bcc strains was constructed by OrthoFinder and compared with the pattern of gene content (Figure 2). The phylogenetic tree revealed a greatly diversified population structure consisting of indistinct lineages with various branch lengths, even for a same species. The distribution of accessory genes also varied among strains as well as clades (Figure 2).

### General Features of the Core Orthologous Genes in the 116 Bcc Genomes

Among the universal core-genome of Bcc, 1005 orthogroups consisted entirely of single copy genes in all strains. This set of genes represents the core genomes of these species well since they shared a common ancestor during speciation. Then, those single copy genes had been deemed to be core orthologous genes, which were adopted in later analysis. The core orthologous genes accounted for 11.11–18.26% of the coding genes in each genome. To determine the relationship of genes with some evolutionary features, we classified these genes into different functional categories using COG annotation and conducted statistical tests.

Through comparison, we found that, there were large numbers of informative sites of genes in the functional categories “cell wall/membrane/envelope biogenesis (M)” and “amino acid metabolism and transport (E)” (both Ps < 0.001 upon one-sided U-test) relative to those in the remaining categories (Table 1).

**TABLE 1:** Relationships of COGs in single copy orthogroups with the descriptive variables.

COG	Function	Number of genes	Bonferroni-corrected *P*-value for one-sided U-test for association							
		analyzed	between genes in a given COG and the others							
			More informative sites	Fewer informative sites	Higher *d*_s_	Lower *d*_s_	Higher *d*_N_	Lower *d*_N_	Higher codon bias	Lower codon bias
E	Amino acid metabolism and transport	87	<0.001			0.024			<0.001	
F	Nucleotide metabolism and transport	55						0.014		
C	Energy production and conversion	68				0.006		0.047	0.005	
M	Cell wall/membrane/envelope biogenesis	51	<0.001							
J	Translation, including ribosomal structure and biogenesis	97		<0.001		<0.001		<0.001		
K	Transcription	82			0.001					<0.001
S	Function unknown	194		0.01	<0.001		<0.001			<0.001
–	Not in COGs	48			<0.001		<0.001			<0.001

The N_c_ value was adopted for measuring codon bias in all core orthologous genes (Peden, 2000). The smaller N_C_ value indicates a greater codon bias, and a significant codon bias is represented by an N_c_ value less than or equal to 35 (Comeron and Aguadé, 1998). The greater codon use bias was seen in “energy production and conversion (C)” and “amino acid metabolism and transport (E)” COG functional categories for Bcc core genes (*P* = 0.005 and *P* < 0.001, separately, upon one-sided U-test) relative to those of the remaining COG categories (Table 1). As reported previously, genes that have more potent codon bias may show high expression and possess the housekeeping characteristics (Gouy and Gautier, 1982; Gharbia et al., 1995; Orsi et al., 2008). Therefore, the markedly increased codon use bias for genes of those two categories may state that it is required for necessary Bcc strain physiological activities and related coding products during the basic life cycle.

*Burkholderia cepacia* complex genes in the functional categories “transcription (K)” might be associated with increased synonymous (*d*_S_) nucleotide substitution rates (*P* = 0.001; one-sided U-test) relative to those of the remaining functional categories. Noteworthily, genes within the “translation, ribosomal structure and biogenesis (J)” and “C” categories had markedly reduced mean *d*_N_ and *d*_S_ values (*P* = 0.047, *P* = 0.006, *P* < 0.001, and *P* < 0.001, separately upon one-sided U-test) compared with those within the remaining categories. The genes in “J” category also had significantly fewer informative sites (*P* < 0.001; one-sided U-test). Genes that take part in translational mechanism are generally subjected to slow evolution and have low *d*_N_ and *d*_S_ values, which may be related to the strict functional and structural constraints during the life process of cells (Jordan et al., 2002; Drummond et al., 2005; Xu et al., 2011). The lower evolution rate of COG functional category J was also indicated by a previous study (Luo et al., 2015). Moreover, except for category “defense mechanisms (V)” within only six genes, for Bcc genes in other COG categories, *d*_S_ value was positively correlated with *d*_N_ value, which suggested the evenly potential effect of natural selection on nonsynonymous as well as synonymous sites for those genes. The genes of the Bcc core genome in each functional category had higher *d*_S_ and *d*_N_ values than the genes of other bacterial core genomes, for example, *E. coli* (Jordan et al., 2002) and *A. pleuropneumoniae* (Xu et al., 2011; Supplementary Table S2), indicating that strains in Bcc might undergo stronger natural selection.

**FIGURE 3:**
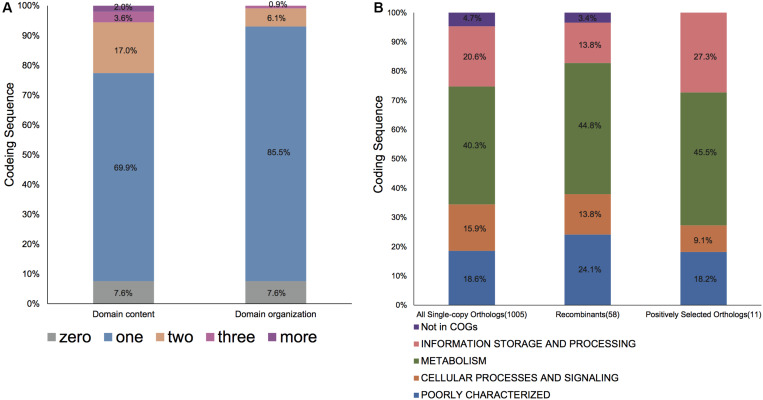
The protein composition and function distribution of single copy genes in Bcc. **(A)** The domain content repertoires and domain organization repertoires distribution of all 1005 orthologous families. The domain content repertoire of an ortholog is defined as the set of domains that occur in the proteins of the orthologous family; domain organization represents the sequential protein domain order; for an ortholog, domain organization repertoire is referred to as a group of domain organizations for each encoded protein within the orthologous family. Note that one ortholog including four different domain organizations was not shown in the figure. **(B)** The COG functional distribution of all single copy genes, evidently recombinant genes, and genes under positive selection. The number of each typical ortholog is given in parentheses. The functional classes are colored as listed in the bottom. Each category is graphed as a percentage of the total number of othologs in the corresponding gene set.

We then explored the domain content and organization of proteins coded by these core orthologous genes. The Pfam-A database was retrieved to annotate each gene (Finn et al., 2014). Of the 1005 core orthologous genes examined, most genes only have a single Pfam domain and more genes are comprized of single domain organization (Figure 3A). We also found seven orthologous families that were composite genes, especially four (OG00011776, *ychA*, S; OG0001404, *glnS*, J; OG0001725, *OmpR*, KL; OG0001325, TonB-dependent receptor, P) that were comprised almost entirely of composite genes.

### Recombination Analysis of Species in Bcc

Various homologous recombination signatures from the 1005 core ortholog families were inspected using four common statistical test methods, including GENECONV (Sawyer, 1989), NSS, Max-χ^2^ and PHI (Bruen et al., 2006). Our analysis identified a total of 787 orthologous core genes (78.3% of all 1005 core genome genes) with evidence for homologous recombination in at least one of these analyses after multiple tests correction (FDR < 10%). Specifically, 202, 413, 624, and 281 orthologs had been recognized to show the recombination signals through PHI, Max-χ^2^ NSS, and GENECONV, separately. In addition, altogether 58, 152, 255, and 322 orthologs had been recognized to show recombination signals through all four, three, two, and one recombination tests, separately.

**FIGURE 4:**
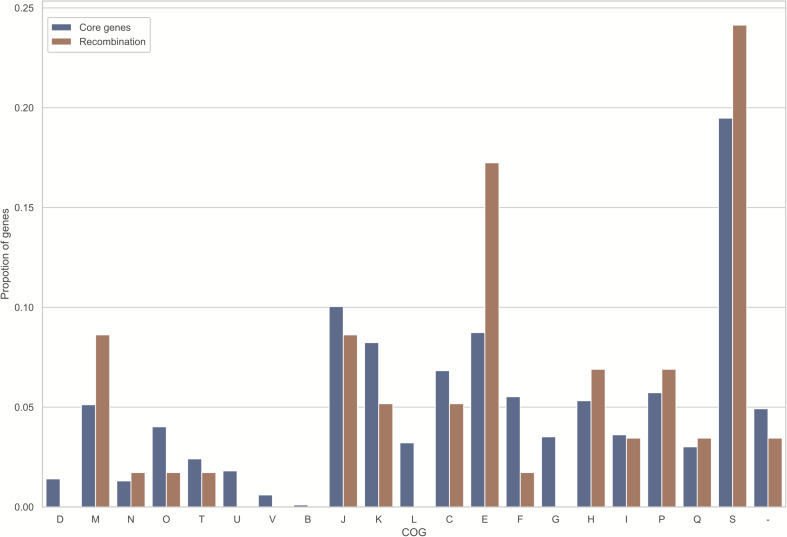
Those homologously recombined genes are distributed across every COG evenly. The X-coordinates stand for the diverse functional categories of COG, while the Y-coordinates stand for gene proportion within every functional category. Meanwhile, the blue and orange bars represent proportion of single copy genes of each COG, and that of recombined genes (FDR < 10%), separately. Meanwhile, those COG categories are shown below: D, chromosome partitioning, cell division, cell cycle control; M, cell envelope/membrane/wall biogenesis; N, cell motility; O, modification at post-transcription level, chaperones and protein turnover; T, mechanisms of signal transduction; U, vesicular transport, secretion and intracellular trafficking; V, mechanisms of defense; B, chromatin dynamics and structure; J, translation, such as biogenesis and ribosomal structure; K, transcription; L, repair, recombination and replication; C, energy conversion and production; E, amino acid transport and metabolism; F, nucleotide transport and metabolism; G, carbohydrate metabolism and transport; H, coenzyme metabolism and transport; I, lipid metabolism and transport; P, inorganic ion metabolism and transport; Q, catabolism, transport, and biosynthesis of secondary metabolites; S, unknown function; –, unknown proteins not collected in COG categories.

Noteworthily, 58 (5.8%) core genome genes (Supplementary Table S3), which were identified to exhibit recombination signals by all four tests, possess a larger number of informative sites (*P* < 0.001 upon one-sided U-test), along with the greater nonsynonymous (*d*_N_) and synonymous (*d*_S_) substitution rates (*P* < 0.001 and *P* = 0.001, respectively, upon one-sided U-test). Unsurprisingly, those orthologs that exhibited recombination signals had significantly longer lengths than those that did not exhibit recombination signals (*P* < 0.001; one-sided U-test). Such finding conforms to the hypothesis that, genes with a lower length may potentially participate in recombination due to the reduced analysis power for shorter recombinant genes and/or lowered target size (Wiuf et al., 2001; Orsi et al., 2008; Dillon et al., 2019). Additionally, the relationship of COG categories with recombination signature-containing gene proportion was predicted (Figure 4). Only the category “E” was significantly overrepresented with recombined genes (uncorrected *P* = 0.028; one-sided binomial test). Nonetheless, all recombinant genes were distributed among various COGs, and there was no significance following multiple comparisons corrected by Bonferroni method. Furthermore, genes with known functions accounted for nearly three-quarters of 58 recombined genes (Figure 3B). Most of them were metabolism-related genes.

**FIGURE 5:**
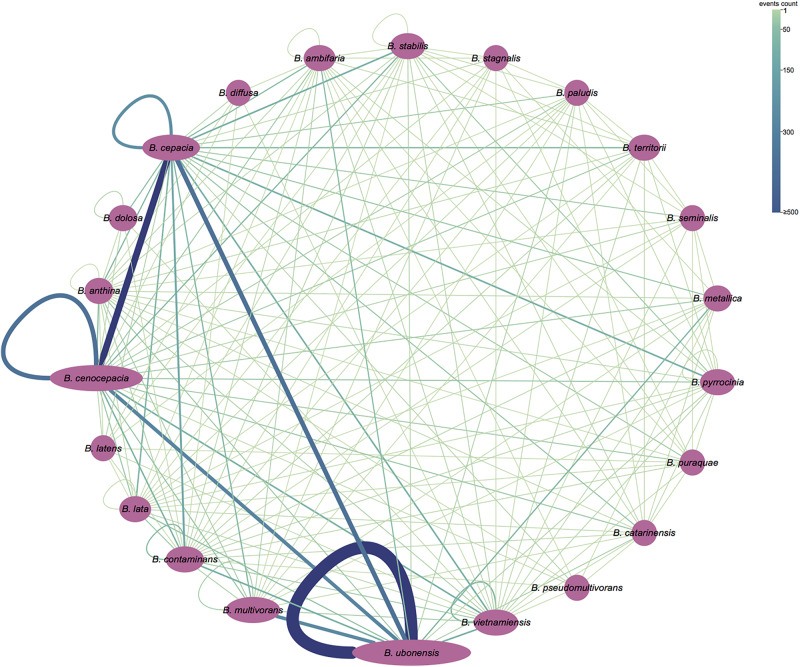
Partition of recombination events (“inner” fragments) detected by GENECONV. The purple ellipse represents the bacterial species in Bcc, and the area of ellipse corresponds to its genome numbers. A line between two ellipses means recombination events between the strains of the corresponding species; a loop line means the recombination events between strains of the same species. The number of recombination events is shown by the width and the color of the line. The figure was visualized and colored by Cytoscape version 3.7.1.

To gain insights into the level of homologous recombination between and within Bcc species, we additionally partitioned the “inner” fragment conversion of results obtained from GENECONV analyses to the inter- and intra-species recombinant events. The “inner” fragment conversions indicate that they are evidence of a possible recombination event between ancestors of two sequences in the alignment. Our results demonstrated that orthologs that recombine between species (3922 events; 67.1%) are more common than orthologs that recombine within species (1925 events; 32.9%) (Figure 5). The most frequent core gene recombination occurred in *Burkholderia ubonensis* (1141 and 1348 intra- and inter-species recombinant events, respectively), followed by 2121 (1882; 239) recombination events in *B. cepacia* and 1806 (1429; 377) recombination events in *B. cenocepacia*.

### Positive Selection Detection on Core Orthologous Genes

For detecting the underlying Bcc genes responding to the host niches, we carried out selection analyses on the basis of LRT of the pairwise evolution models (M1a vs. M2a, as well as M7 vs. M8, site-model) for 1005 single copy core genes of Bcc, followed by multiple tests (FDR < 10%). The M7 vs. M8 LRT identified 101 genes (10.1%) under positive selection, while M1a vs. M2a LRT, which was more conservative, recognized 12 (1.2%) positively selected genes. The evolved Bcc single copy gene proportion screened at positive pressure through the use of M8 model was similar to those detected within *Burkholderia oklahomensis*, *Burkholderia thailandensis*, *Burkholderia pseudomallei* and *Burkholderia mallei* clade (10.6%, 197/1842) (Bochkareva et al., 2018). Those 11 (1.1%) shared genes had been screened positively through M1a vs. M2a as well as M7 vs. M8 LRTs, and they were held in later analyses for robustness. Of the 11 positively selected genes, all but two were categorized as “function unknown (S),” two belonged to category “J,” while two were in the “inorganic ion transport and metabolism (P)” category. As for those five residual genes, they were divided into “E,” “V,” “secondary metabolite biosynthesis, transport, and catabolism (Q),” “K,” and “coenzyme transport and metabolism (H)” categories (Table 2). No COG categories were significantly overrepresented among these 11 genes, since there are few genes screened positively within every functional category. In addition, there was no obvious discrepancy for properties between positively selected genes and those residual genes. According to subcellular locations of proteins, approximately one-quarter (3/11) of genes under positive selection encoded products that were located onto cell membrane and with transmembrane alpha helix structure (Table 2).

**TABLE 2:** Genes under positive selection.

Cluster ID	Gene	COG	Function	Positively selected sites (P_BEB_ ≥ 0.95)	ω_(M2a)_	*q*-Value from M1a vs. M2a (FDR < 10%)	Domain (Pfam)	Subcellular localization	Feature
OG0001459	*selU*	H	tRNA 2-selenouridine synthase	363	7.966	4.77E-06	–	Cytoplasm	Unknown
OG0001809	–	S	Conserved hypothetical protein	91	4.522	0.00019	DUF2889 (PF11136)	Cytoplasm	Unknown
OG0001972	*gatB*	J	Aspartyl/glutamyl-tRNA_Asn/Gln_ amidotransferase subunit B	119	4.479	0.00097	GatB/GatE catalytic domain (PF02934)	Cytoplasm	Unknown
OG0001276	*ybgC*	S	4-hydroxybenzoyl-CoA thioesterase	155	5.109	0.00097	–	Cytoplasm	Unknown
OG0002150	–	P	Flavin-containing monooxygenase FMO	210	4.047	0.00227	Flavin-binding monooxygenase-like (PF00743)	Cytoplasm	Unknown
OG0001150	*yadH*	V	Transport permease protein	31, 35	4.289	0.00697	ABC2_membrane (PF01061)	Plasma membrane	Transmembrane alpha helix
OG0001116	*rplE*	J	Ribosomal protein L5	94	6.183	0.03080	Ribosomal_L5_C (PF00673)	Cytoplasm	Unknown
OG0001513	–	K	MerR family regulatory protein	7, 137	3.437	0.04362	–	Cytoplasm	Unknown
OG0001392	*leuE*	E	leucine efflux protein	108, 125	2.681	0.04704	LysE (PF01810)	Plasma membrane	Transmembrane alpha helix
OG0001464	*cysW*	P	Sulfate ABC transporter, inner membrane subunit CysW	301, 311	3.96	0.06831	–	Plasma membrane	Transmembrane alpha helix
OG0002263	–	Q	Dopa 45-dioxygenase	109	3.672	0.06880	DOPA_dioxygen (PF08883)	Cytoplasm	Unknown

*The *q*-values denote the Benjamini and Hochberg corrected *P*-values from M1a Vs M2a LRT (FDR < 10%). The “–” in the domain column means that positively selected sites are not located on any domain region. Positively selected sites identified with posterior probability *P* ≥ 95%, based on M1a Vs M2a. ω represents d_N_/d_S_ ratio for those positively selected amino acid sites (model M2a).*

**FIGURE 6:**
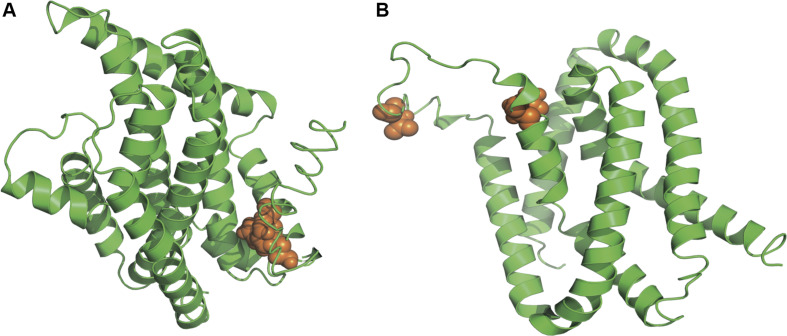
Three-dimensional structural models of material transport proteins *yadH* **(A)** and *leuE/lysE* **(B)**. Orange spheres stand for amino acid residues that are subject to strong positive selection (BEB posterior probability ≥95%).

**FIGURE 7:**
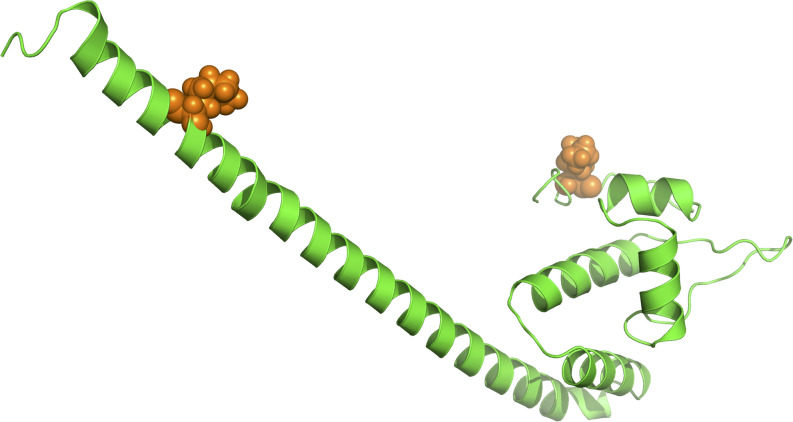
Three-dimensional structural models for one transcriptional regulator in the MerR family. The orange spheres represent the positively selected amino acid residues (BEB posterior probability ≥95%).

As suggested in this study, those two genes (*CysW* and *yadH*) products, which were predicted to be cytoplasmic membrane component and permease component of ATP-binding cassette (ABC) transporter, respectively, exhibited significant evidence of positive selection. According to BEB analysis, in those two genes, the two amino acid residues were positively selected (Table 2). Each of the *yadH* residue detected was localized on the identical α-helix (Figure 6A). One amino acid transport gene, *leuE*/*lysE*, which was predicted to mediate the efflux of leucine and/or lysine (pairwise amino acid identity of sequences in this family is >90%), was identified with evidence for positive selection. It is noteworthy that one of two positively selected sites is located on a transmembrane α-helix (Figure 6B). Another positively selected gene is flavin-containing monooxygenase FMO, which was predicted to be involved in bacterial trimethylamine (TMA) metabolism and other carbon as well as nitrogen cycle (Chen et al., 2011; Ceccoli et al., 2014). Two genes (*ybgC* and MerR family transcriptional regulator) involved in response to survival stress showed significant evidence for positive selection. *ybgC* proteins are part of the *Tol-Pal* system that maintains cell membrane integrity (Angelini et al., 2008; Qin et al., 2019). It was also shown to be linked to bacterial motility (Gao et al., 2017), affect the *Tol-Pal* system and play a crucial role in *S*almonella *enteritidis* survival under egg white stress (Qin et al., 2019). It is reported that, the transcriptional regulator in the MerR family can respond upon the various environmental stimuli, including antibiotics, heavy metals, and oxidative stress (Brown et al., 2003; Ghosh et al., 2017). A predicted model of the MerR-like protein showed that positively selected residues are located on a loop around the N-terminal helix-turn-helix DNA binding regions, and another is located on the last α-helix (Figure 7). *selU*, the tRNA 2-selenouridine synthase, enables the conversion of S2U-RNA into Se2U-RNA through the intermediates obtained from S-geranylation (Sierant et al., 2018), which strongly supports strong positive selection and has a low *q*-value (Table 2). Another positively selected gene, *gatB*, encodes subunit B of the heterotrimeric amidotransferase required for producing correctly aminoacylated Gln-tRNA^Gln^ and Asn-tRNA^Asn^ during translation (Curnow et al., 1997; Akochy et al., 2004). It was shown that *gatB* played an essential role in maintaining the fidelity of the genetic code (Shepherd and Ibba, 2015) and possibly in generating phenotypic diversity (Su et al., 2016).

### Summary of Soft-Core Gene Orthogroups Analysis Results

To improve the credibility of our conclusions derived from single copy core genome analysis, additional 770 orthogroups in the soft-core genome were brought into analysis (see section “Materials and Methods”). Firstly, our comparison result showed that genes in the functional categories “C” and “E” had larger numbers of informative sites than those in the remaining categories (*P* = 0.003 and *P* = 0.014, upon one-sided U-test). In addition to the functional categories “E” and “C” like in single copy orthologous groups, the genes in category “nucleotide metabolism and transport (F)” also exhibited higher codon bias (*P* = 0.024, *P* = 0.031, and *P* = 0.010, respectively from one-sided U-test). As regards substitution rates, similar to single copy orthologous groups, only genes in the functional category “K” was might be associated with increased *d*_S_ (*P* = 0.041, one-sided U-test). The genes in categories “F” and “P” owned notably reduced mean *d*_S_ values (both *P* < 0.001, one-sided U-test), while those in categories “F” and “K” showed notably reduced mean *d*_N_ values (*P* < 0.001 and *P* = 0.017, respectively, one-sided U-test) (Supplementary Table S4). What is more, mean *d*_S_ value was positively correlated with mean *d*_N_ value in all categories of the soft-core genes.

Secondly, a total of 94 (12.2%) soft-core genes were identified to show evidence for recombination by all four methods after multiple test correction (FDR < 10%). These genes exhibited more number of informative sites (*P* < 0.001), longer length (*P* < 0.001), increased mean *d*_N_ values (*P* < 0.001), and higher codon usage bias (*P* = 0.005, one-sided U-test). Among them, genes were significantly overrepresented in functional category “M” (Bonferroni-corrected *P* = 0.028, one-sided binomial test) (Supplementary Figure S1). Specifically, GENECONV analysis results demonstrated that there were more inter-species recombination events (4302; 69.8%) than intra-species (1861; 30.2%) in soft-core genome of Bcc.

Thirdly, conservative M1a vs. M2a LRT identified 16 genes under positive selection (FDR < 10%) among the soft-core orthogroups. No COG categories were significantly overrepresented and no remarkable discrepancy for properties was observed among the positively selected genes. Similarly, most of them were metabolism-related genes (Supplementary Figure S2). Subcellular locations predication suggested one-quarter (4/16) of genes under positive selection encoded products that were located on cell membrane/extracellular space.

## Discussion

In this study, we performed a comprehensive analysis on the genomes of a closely related but diverse collection of 116 Bcc strains comprising 22 species to gain insight into the core genome characterization and evolutionary dynamics of the *B. cepacia* complex.

The Bcc pan-genome is immense and divergent, including a grand total of 17,740 otholog families. Yet, more than half of them are present at less than 15% of Bcc strains. Our analyses suggested the pan-genome of Bcc is open and more novel genes might be discovered with additional sequenced genomes in the future. Gene accumulation curves demonstrates that the size of core genome stabilizes after sampling around 110 genomes at ∼1400 genes. Specifically, a total of 1005 orthologous single copy genes had been identified present in all strains, which highly approximates the size of the core genome of other *Burkholderia* spp. (Ussery et al., 2009; Bochkareva et al., 2018). We classified these genes into different COG categories and conducted statistical tests between genes and several evolutionary properties. Orthologous genes in some COG categories showed significantly higher or lower informative sites, codon bias and evolutionary rates (*d*_S_ or *d*_N_) than others. Pfam annotations suggested that proteins encoded by the Bcc core genome were relatively simple and compact. The results showed a large portion (69.9%) of these orthologous genes coded single domain proteins, and most (85.5%) orthologous families contained only a single domain organization. In addition, the results showed that some orthologous families comprising partial or whole members were fused genes.

Detection of intragenic homologous recombination with 1005 single copy orthologous genes of Bcc revealed that 5.8% of genes in the Bcc core genome showed significant signatures of homologous recombination. Other species also displayed obvious recombination of core genomes. In contrast, 6.3% core genomes distributed among four *E. coli* as well as two *Shigella* genomes showed strong proof of recombination (Petersen et al., 2007); analysis of 33 *P. multocida* genomes found that approximately 7% of single copy core genomic genes showed the recombination proof (Cao et al., 2017). Additionally, 23 and 62.7% of core genome genes for *A. pleuropneumoniae* (Xu et al., 2011) and *Neisseria* spp. (Yu et al., 2014), respectively, were identified to show significant signatures of homologous recombination. Importantly, our analysis suggested that orthologs that recombine between species are more common than orthologs that recombine within species in Bcc. Recombination analysis of soft-core genes supported the conclusion as well. In addition, homologous recombination occurred in every functional category evenly. These phenomena demonstrated that homologous recombination could be a crucial force for maintaining extensive genetic cohesion in Bcc and enhancing the intimate similarity of species in Bcc, which was also observed in other bacteria species complex (Dillon et al., 2019).

Eleven genes of Bcc, approximately 1.1% of the single copy orthologous genes, were identified being subjected to positive selection. A similar proportion was also observed in *Neisseria* (Yu et al., 2014) and *Salmonella* core genomes (Soyer et al., 2009) but decreased relative to additional pathogenic bacteria, including *P. multocida* (Cao et al., 2017) and *A. pleuropneumoniae* (Xu et al., 2011). Except for one hypothetic protein, many Bcc genes under positive selection pressure take part in material metabolism and transport, stress response and protein synthesis.

First, bacterial ABC transporters, including importers, exporters, and systems that are involved in the translation of mRNA and in DNA repair, are crucial to cell viability, virulence, and pathogenicity (Davidson et al., 2008; El-Awady et al., 2017). We found that two components of the ABC transporter, *CysW* and *yadH*, were favored by selection pressure. The former constitutes an ABC importer that is required for sulfate and thiosulfate import (Sirko et al., 1990; Farmer and Thomas, 2004), and the latter constitutes an ABC-type multidrug transport system. Another leucine and/or lysine transport gene, *leuE*/*lysE*, was also identified as subject to positive selection. In addition to transmembrane transport genes, flavin-containing monooxygenase FMO involved in multiple metabolic cycles was found under positive selection as well. There are always high sputum contents of amino acids among the CF cases, in particular following severe bacterial infections (Barth and Pitt, 1996; Thomas et al., 2000; Mira et al., 2011), positive selection of these transport- and metabolism-related genes contributes to reflecting long-time Bcc strain adaptation to the specific CF lung nutritional environment. In addition, *yadH* shared over 90% amino acid identity with *BCAL0308*, which may be part of the Mla pathway in *Burkholderia* and possibly interact with the other Mla proteins, participating in the intrinsic resistance of *Burkholderia* to Gram-positive antibiotics (Bernier et al., 2018). Selection pressure on *yadH* may also be related to the significant antibiotic resistance of Bcc. Second, two positively selected genes, *ybgC* and the MerR family transcriptional regulator, were involved in the stress response. Environmental stress detrimentally affect the viability of cells, and the sufficient cell activity reprogramming is necessary to achieve the maximal cell survival (Bartholomäus et al., 2016). Positive selection on these genes might result from a need for rapid adaptation to the ever-changing environmental conditions; for example, the increasing antibiotic use in clinical treatments of CF patients. Third, three translation-related genes, *selU, gatB*, and *rplE*, exhibited evidence for positive selection. It is known that those modified nucleosides that are observed within the transferred RNAs can dynamically regulate expression of genes and control translation of proteins, as a result, cells can quickly react to changes in environment (such as various stress); besides, the ability to synthesize proteins can be used to deliver the most essential proteins (Sierant et al., 2016, 2018; Lorenz et al., 2017).

Interestingly, 10 of 11 positively selected genes were identified to have signatures of homologous recombination by at least one test (Supplementary Table S3). Among them, two displayed obvious recombination evidence, as verified through those four recombination tests (Supplementary Table S3). Such findings demonstrated the possible relationship between positive selection and the intragenic recombination. In addition, positive selection potentially play a vital role in maintaining the recombination-introduced fragments for the given population, when they are selectively advantageous for recipient organism. Furthermore, recombination may also result in phylogenetic incongruence, thereby causing false positiveness when the selection pressure was estimated on those protein encoding sequences (Anisimova et al., 2003; Orsi et al., 2008; Xu et al., 2011).

## Conclusion

In summary, our study defined the single copy core genome of Bcc species and its general characteristics as well as the underlying adaptive evolutionary forces. Through analysis, we estimated 1005 single copy orthologous genes that were used to represent the core genome of Bcc. Our results showed that genes in some COG categories showed significant differences in the comparison of several evolutionary properties, and the encoding proteins were relatively simple and compact. Our findings indicated that the evolutionary dynamics of the single copy core genome of Bcc are driven by both homologous recombination and positive selection. It is an important that recombination between species is more common than within species of Bcc. This high level and evenly occurring recombination between Bcc species largely maintained the genetic cohesion in Bcc and blurred their taxonomic boundaries, which led Bcc species to be difficult or impossible to distinguish phenotypically and genotypically. We also found that genes involved in protein synthesis as well as material transport and metabolism are favored by positive selection pressure. These positively selected genes might serve as the targets to further researches on the adaption mechanism and the host-pathogen interactions within Bcc.

## Data Availability Statement

All genomes were downloaded from NCBI GenBank, and detailed accession numbers could be retrieved from Supplementary Table S1.

## Author Contributions

JY, LL, and YJ formulated the study. HR and JiaZ performed the research. MH, JinZ, BL, NK, and QZ analyzed the data. HR, BL, and LL participated in the analysis, discussion, and support. JiaZ and YJ drafted the manuscript. JiaZ, JY, and YJ revised the manuscript. All authors read and approved the final manuscript.

## Conflict of Interest

The authors declare that the research was conducted in the absence of any commercial or financial relationships that could be construed as a potential conflict of interest.

## Footnotes

1 http://www.ncbi.nlm.nih.gov/genome

2 http://codonw.sourceforge.net

3 http://eggnog-mapper.embl.de/

4 http://www.pymol.org

## Supplementary Material

FIGURE S1 Genes with signal of recombination are enriched in the COG functional category “M.” The X-coordinates stand for the diverse functional categories of COG, while the Y-coordinates stand for gene proportion within every functional category. Meanwhile, the blue and orange bars represent proportion of soft-core genes of each COG, and that of recombined genes (FDR < 10%), separately. Asterisks mark certain COG category that significantly enriched with recombining genes (Bonferroni-corrected *P* < 0.05, binomial test). The abbreviations of those COG categories are same as shown in the Figure 4 legend.

FIGURE S2 The COG functional distribution of soft-core genes, evidently recombinant genes, and genes with under positive selection. The number of each typical ortholog is given in parentheses. The functional classes are dyed as listed in the bottom.

TABLE S1 Metainformation table of genomes used in this study. The quality estimates (completeness, contamination) of these genomes were determined with CheckM using the lineage-specific workflow and default parameters. The quality of a genome was defined as its completeness – 5 × contamination.

TABLE S2 Nonsynonymous (*d*_S_) as well as synonymous (*d*_N_) nucleotide substitution rates of single copy core Bcc genes of various functional categories.

TABLE S3 The table of orthologous core genes with recombination signals in at least one of the four detection methods.

TABLE S4 Relationships of COGs in soft-core orthogroups with the descriptive variables.
