# Supplementary figures and images for: Characterization of Burkholderia cepacia Complex Core Genome and the Underlying Recombination and Positive Selection
Source: Front Genet. 2020 May 21;11:506. doi: 10.3389/fgene.2020.00506 (PMC7253759; doi:10.3389/fgene.2020.00506)

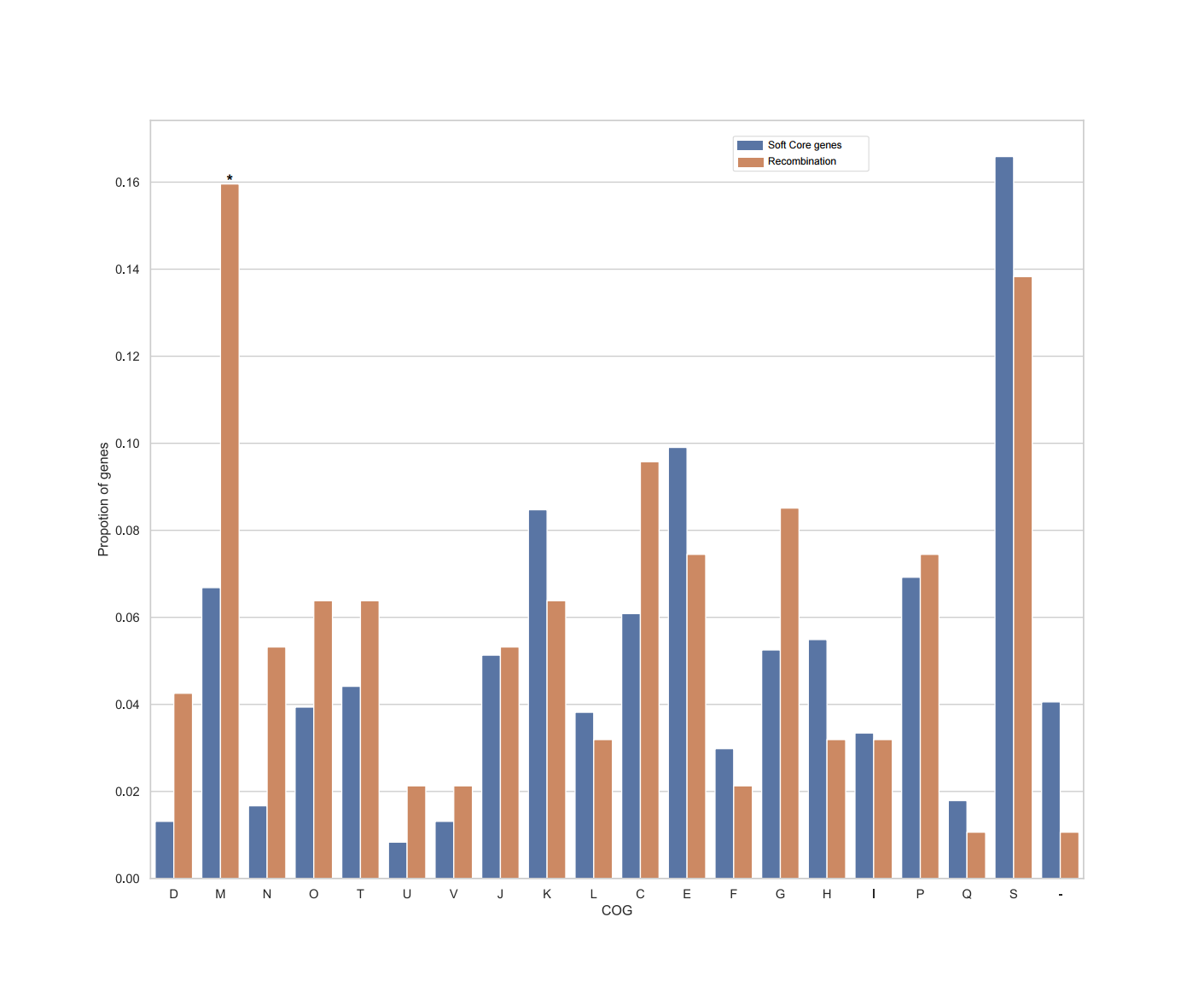

Supplement: FIGURE S1 — Genes with signal of recombination are enriched in the COG functional category “M.” The X-coordinates stand for the diverse functional categories of COG, while the Y-coordinates stand for gene proportion within every functional category. Meanwhile, the blue and orange bars represent proportion of soft-core genes of each COG, and that of recombined genes (FDR < 10%), separately. Asterisks mark certain COG category that significantly enriched with recombining genes (Bonferroni-corrected P < 0.05, binomial test). The abbreviations of those COG categories are same as shown in the Figure 4 legend. [file Image_1.TIFF]

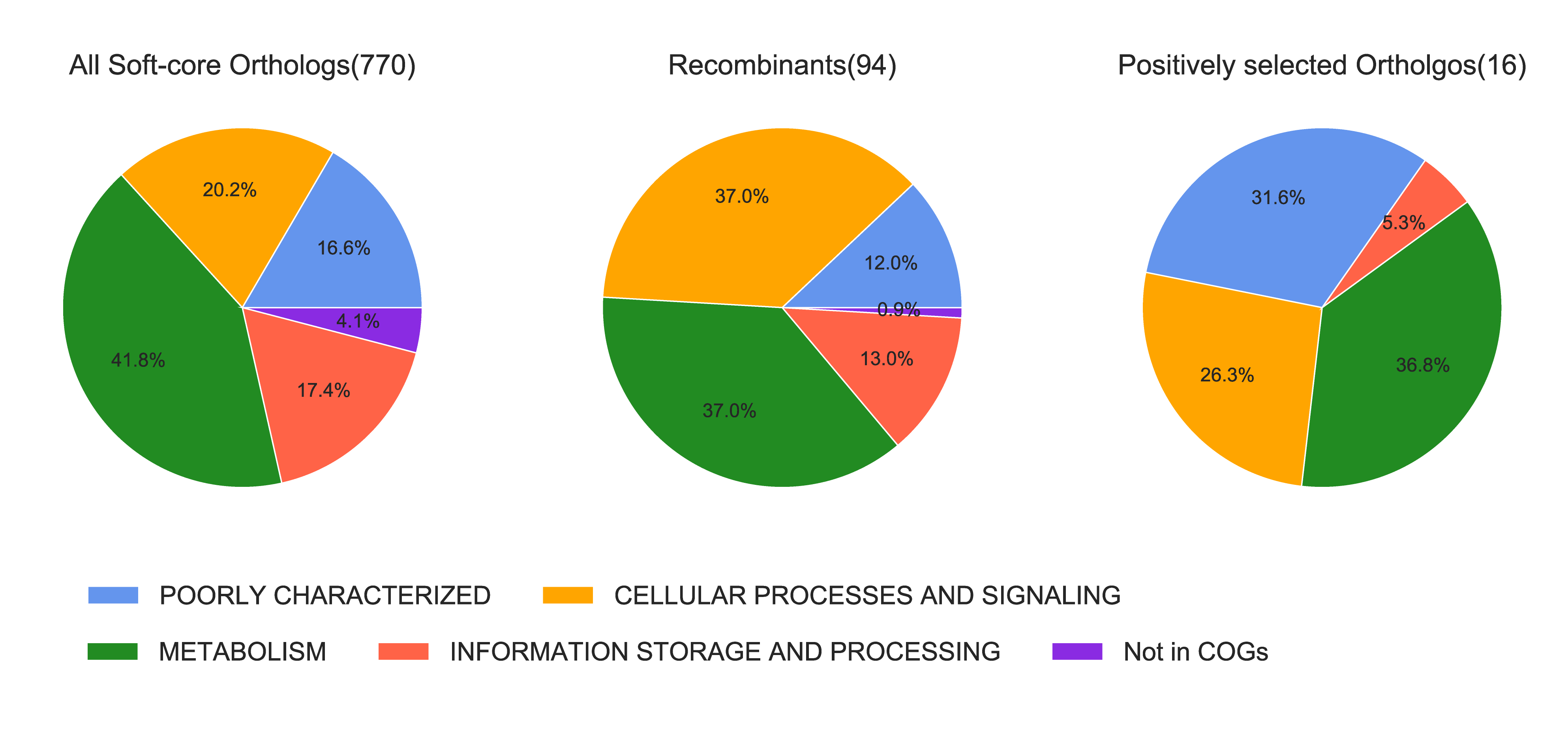

Supplement: FIGURE S2 — The COG functional distribution of soft-core genes, evidently recombinant genes, and genes with under positive selection. The number of each typical ortholog is given in parentheses. The functional classes are dyed as listed in the bottom. [file Image_2.TIFF]
